# Chemical Reactivity Properties, p*K*_a_ Values, AGEs Inhibitor Abilities and Bioactivity Scores of the Mirabamides A–H Peptides of Marine Origin Studied by Means of Conceptual DFT

**DOI:** 10.3390/md16090302

**Published:** 2018-08-28

**Authors:** Juan Frau, Norma Flores-Holguín, Daniel Glossman-Mitnik

**Affiliations:** 1 Departament de Química, Universitat de les Illes Balears, 07122 Palma de Mallorca, Spain; juan.frau@uib.es; 2 Laboratorio Virtual NANOCOSMOS, Departamento de Medio Ambiente y Energía, Centro de Investigación en Materiales Avanzados, Miguel de Cervantes 120, Complejo Industrial Chihuahua, Chihuahua, 31136 Chih, Mexico; norma.flores@cimav.edu.mx

**Keywords:** Mirabamides A–H, computational chemistry, conceptual DFT, chemical reactivity, bioactivity scores

## Abstract

The MN12SX density functional, in connection with the Def2TZVP basis set, was assessed, together with the SMD solvation model (Solvation Model based on the Density), for calculation of the molecular properties and structure of a group of peptides of marine origin named Mirabamides A–H. All the chemical reactivity descriptors for the systems were calculated via Conceptual Density Functional Theory (CDFT). The active sites suitable for nucleophilic, electrophilic, and radical attacks were chosen by linking them with the Fukui function indices, nucleophilic and electrophilic Parr functions, and condensed Dual Descriptor Δf(r), respectively. Additionally, the p$K_{a}$ values for the different peptides are predicted with great accuracy as well as the ability of the studied molecule in acting as an efficient inhibitor of the formation of Advanced Glycation Endproducts (AGEs), which constitutes a useful knowledge for the development of drugs for fighting Diabetes, Alzheimer and Parkinson diseases. Finally, the bioactivity scores for the Mirabamides A–H are predicted through different methodologies.

## 1. Introduction

The sea is an inexhaustible source of natural resources that give rise to molecules that can serve as a guide for the development of new medicines. For this reason, numerous investigations have been carried out in recent years dedicated to the search for new natural products that can be obtained from the knowledge of marine species [1].

Among the chemical species that can be obtained from natural products of marine origin, the peptides that are molecules of intermediate size between amino acids and proteins stand out. The therapeutic application of these peptides, called for this reason therapeutic peptides, is currently one of the most active fields of research due to the great possibilities they represent as aids for the treatment of numerous diseases [2].

For the consideration of therapeutic peptides from the point of view of medicine, it is necessary to know their molecular properties and their bioactivity. It is our belief that the bioactivity of these peptides is intimately related to their chemical reactivity from a molecular perspective [3,4]. For this reason, we consider it essential to study the chemical reactivity of natural products that have the potential to become medicines through the tools provided by Computational Chemistry and Molecular Modeling. The most powerful tool currently available to study the chemical reactivity of molecular systems from the point of view of Computational Chemistry and Molecular Modeling is probably the Conceptual DFT (Density Functional Theory) [5,6], also called Chemical Reactivity Theory, which, using a series of global and local descriptors, allow the prediction of the interactions between molecules and the understanding of the way that chemical reactions proceed [7,8,9].

Considering that the knowledge of the chemical reactivity is essential for the development of new medicines from natural products, we have decided in this work to study a series of peptides obtained from marine sponges that have been recently isolated and characterized, and that could be the foundation of new therapeutic peptides [10,11].

The objective of this work is to study the chemical reactivity of the Mirabamides A–D [10] and the Mirabamides E–H [11] using the techniques of the Conceptual DFT, determining its global properties (of the molecule as a whole) as well as the local properties that allow to understand and predict active reaction sites, both electrophilic and nucleophilic. Similarly, the p$K_{a}$ values for each of the peptides will be predicted based on a methodology previously developed by us [12], the ability of these therapeutic peptides to act as inhibitors of the formation of Advanced Glycation Endproducts (AGEs) will be established according to our previous ideas [13], and the descriptors of bioavailability and bioactivity (Bioactivity Scores) will be calculated through different procedures described in the literature [14,15].

## 2. Theoretical Background

As this work is a part of an ongoing study related to our project on Computational Medicinal Nanochemistry, the theoretical background will be similar to that presented in previous works [16,17,18,19,20,21,22] and will be shown here again for completeness reasons. As in those previous works, we will be using the Kohn–Sham theory, which involves the calculation of the molecular density, energy of the system, and the orbital energies particularly associated with the frontier orbitals, including the Highest Occupied Molecular Orbital (HOMO) and Lowest Unoccupied Molecular Orbital (LUMO) [23,24,25,26]. This theory is necessary for establishing the quantitative values of the various Conceptual DFT descriptors. Recently, there has been an increased interest in using range-separated (RS) exchange correlation functionals in Kohn–Sham DFT [27,28,29,30]. These functionals tend to partition the **r**${}_{12}^{-1}$ operator and exchange the parts into long- and short-ranged parts, whose range separation parameter, ω, controls the rate of attaining the long-range behavior. It is possible to fix the value of ω or “tune” it by a system-by-system mechanism that minimizes a tuning norm. The basis of the optimal tuning approach is the fact that the energy of the HOMO, $\epsilon_{H}$(*N*) in case of the exact Kohn–Sham (KS) theory as well as generalized KS theory for an N electron system, should be −IP(*N*). Here, IP represents the vertical ionization potential, which is calculated as the energy difference, E(*N*− 1) − E (*N*), by considering a particular functional. If approximate functionals are used, it could possibly lead to considerable differences between $\epsilon_{H}$(*N*) and −IP(*N*). Optimal tuning involves determining the system-specific range-separation parameter, ω, non-empirically with an RSE functional. Alternatively, it also implies that several other parameters, including $\epsilon_{H}$(*N*) = −IP(*N*), are optimally satisfied [31,32,33,34,35,36,37,38]. Even though there is no equivalent form to match this prescription for deriving the electron affinity (EA) together with the LUMO in case of neutral species, it is possible to say that $\epsilon_{H}$(*N* + 1) = −EA(*N*), which facilitates obtaining the optimized value of ω, and which is then optimized to establish both properties. This would make it easy to predict the Conceptual DFT descriptors. In the past, a simultaneous prescription, referred to as the “KID procedure” owing to its correspondences with the Koopmans’ theorem, was proposed by the authors in References [16,17,18,19,20,21,22]. As it has been explained in the last referenced works, KID stands for “Koopmans in DFT” and is a procedure to check the verification of the $\epsilon_{H}$(*N*) = −IP(*N*) satisfaction and, at the same time, a comparison between the $\epsilon_{L}$(*N*) of the neutral species (the LUMO) and the $\epsilon_{H}$(*N* − 1) for the anionic system (the SOMO or Singly Occupied Molecular Orbital).The descriptor related to this comparison is called ΔSL because is related to the difference between the energies of the SOMO and the LUMO [16,17,18,19,20,21,22].

## 3. Settings and Computational Methods

This study obtained the molecular structure of the Mirabamides A–H peptides from PubChem (https://pubchem.ncbi.nlm.nih.gov), a website that serves as the public repository for information pertaining to chemical substances, along with their associated biological activities. The pre-optimization of the resultant system involved selecting the most stable conformers. The selection was done using random sampling that involved molecular mechanics techniques and the inclusion of the various torsional angles via the general MMFF94 force field [39,40,41,42,43] involving the Marvin View 17.15 program (ChemAxon, Budapest, Hungary), which constitutes an advanced chemical viewer suited to multiple and single chemical queries, structures, and reactions (https://www.chemaxon.com). After that, the chemistry of the structures was checked and the 3D structures of the stereoisomers were generated using the same MarvinView 17.15 program. The chirality at the stereogenic centers was verified in accordance to the Cahn-Ingold-Prelog priority rules. The resulting geometries were further refined, as was previously explained, and the lowest energy conformation for each molecule was chosen to calculate the electronic energy and the HOMO and LUMO orbitals at the DFT functional level, as mentioned in the next paragraph.

Consistent with our previous work [16,17,18,19,20,21,22], the computational studies were performed with the Gaussian 09 [44] series of programs (Gaussian Inc., Wallingford, CT, USA) that implement density functional methods. The basis set Def2SVP was used in this work for geometry optimization and frequency determination, while the Def2TZVP basis set was used for calculating electronic properties [45,46]. All calculations were performed in the presence of water as the solvent under the Solvation Model Density (SMD) parameterization of the Integral Equation Formalism-Polarized Continuum Model (IEF-PCM) [47].

To calculate the molecular structure and properties of the studied systems, we have chosen the MN12SX density functional which is known to consistently provide satisfactory results for several structural and thermodynamic properties [48].

The SMILES (Simplified Molecular Input Line Entry Specification) notations of the studied compounds were fed in the online Molinspiration software from Molinspiration Cheminformatics (Slovensky Grob, Slovak Republic) (www.molinspiration.com) for the calculation of the molecular properties (Log P, Total polar surface area, number of hydrogen bond donors and acceptors, molecular weight, number of atoms, number of rotatable bonds, etc.) and for the prediction of the bioactivity score for different drug targets (GPCR ligands, Kinase inhibitors, Ion channel modulators, Enzymes and Nuclear receptors). The bioactivity scores were compared with those obtained through the use of other software, such as MolSoft from Molsoft L.L.C. (San Diego, CA, USA) (http://molsoft.com/mprop/) and ChemDoodle Version 9.02 from iChemLabs L.L.C. (Richmond, VA, USA) (www.chemdoodle.com).

## 4. Results and Discussion

**Figure 1:**
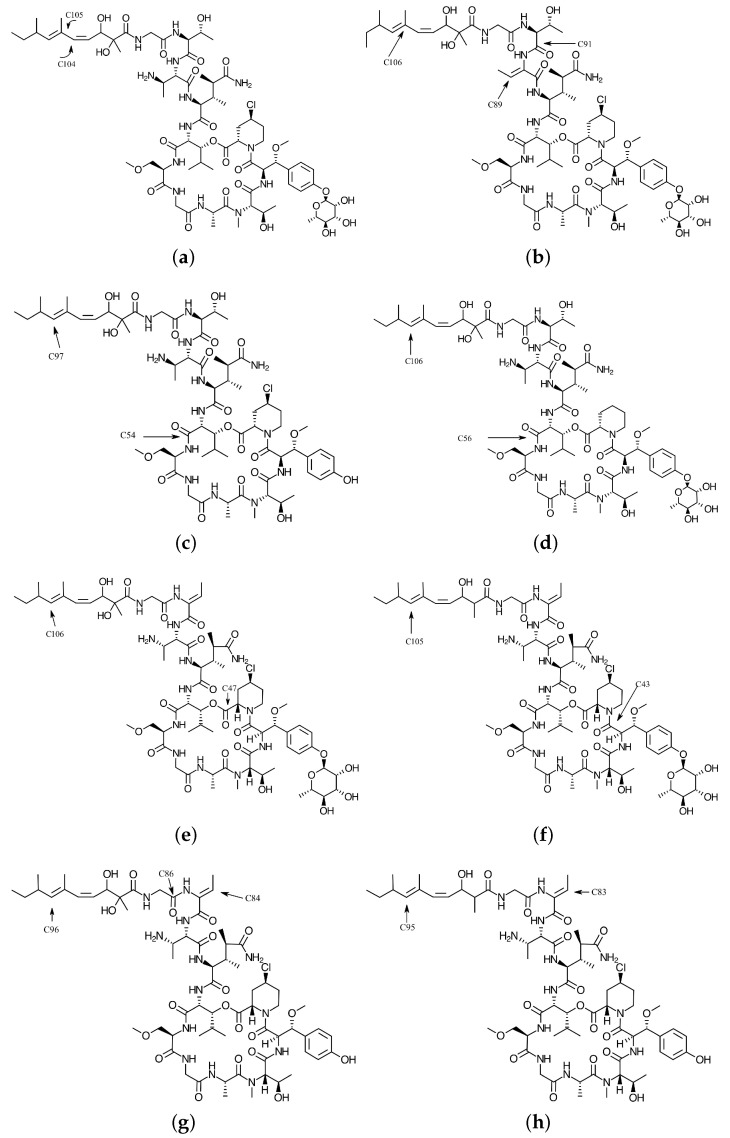
Graphical sketches of the molecular structures (**a**) Mirabamide A; (**b**) Mirabamide B; (**c**) Mirabamide C; (**d**) Mirabamide D; (**e**) Mirabamide E; (**f**) Mirabamide F; (**g**) Mirabamide G and (**h**) Mirabamide H—The numbered arrows indicate the atomic reactive sites according to Tables 3–10.

The molecular structures of the optimized conformers of the Mirabamides A–H obtained as mentioned in the Settings and Computational Methods section, and whose graphical sketches are shown in Figure 1, were reoptimized in the gas phase by considering the DFTBA (Density Functional Tight-Binding Approximation) model available in Gaussian 09 and then optimized again using the MN12SX density functional mentioned in the previous section together with the Def2SVP basis set and the SMD solvent model, using water as the solvent. After verifying that each of the structures corresponded to the minimum energy conformations through a frequency calculation analysis, the electronic properties were determined by using the same model chemistry, but with the Def2TZVP basis set instead of that used for the geometry optimization.

These peptides present a unique structural arrangement that comprises a macrocyclic region closed through an ester bond between the C-terminus and a β-hydroxyl group, and terminated with a polyketide moiety or a more simple branched aliphatic acid [49]. As mentioned in the previous referenced study, two unknown building blocks were described after their isolation: The glycosylated amino acid β-methoxytyrosine 4′-o-α-l-rhamnopyranoside and the 4-chloro-l-homoproline. Thus, these are highly modified glycosylated depsipeptides whose amino acid sequences are hard to explain. However, the sequences for Mirabamides A, C and D starting from the C terminal can be described as: ClHPr-βOMeTyr-NMeThr-Ala-Gly-3-OMeAla-3OHLeu-3,4-DiMeGln-Dab-Thr-Gly-Dhtda. The differences between these are due to the presence or not of the Cl at the terminal proline (substituted by an H) or the rhamnose substitued by an H in Mirabamide C. In the case of Mirabamide B, the sequence is the same as that for Mirabamide A, with the difference of the presence of the Aba instead of the Dab (2,3-diaminobutanoic) residue. For the Mirabamides E–H, the sequence is very similar to that of Mirabamide A, with the only difference being the replacement of threonine with its dehydration product 2-amino-2-butenoic acid (Aba), the replacement of an OH by an H in the polyketide terminal chain and a different conformation for the rhamnose moiety.

The analysis of the results obtained in the study aimed at verifying that the KID procedure was fulfilled. On performing it previously, several descriptors associated with the results that the HOMO and LUMO calculations obtained are related with results obtained using the vertical I and A following the ΔSCF procedure. A link exists between the three main descriptors and the simplest conformity to the Koopmans’ theorem by linking $\epsilon_{H}$ with −I, $\epsilon_{L}$ with −A, and their behavior in describing the HOMO–LUMO gap as $J_{I}=\left|\epsilon_{H}+E_{gs}\left(N-1\right)-E_{gs}\left(N\right)\right|$, $J_{A}=\left|\epsilon_{L}+E_{gs}\left(N\right)-E_{gs}\left(N+1\right)\right|$, and $J_{HL}=\sqrt{{J_{I}}^{2}+{J_{A}}^{2}}$. Notably, the $J_{A}$ descriptor consists of an approximation that remains valid only when the HOMO that a radical anion has (the SOMO) shares some similarity with the LUMO of the neutral system. Consequently, we decided to design another descriptor, ΔSL, to guide in verifying the accuracy of the approximation [16,17,18,19,20,21,22]. The results of this analysis are presented in Table 1.

As can be seen from Table 1, the results for the descriptors show values that are consistent with our previous findings for the case of the melanoidins [16,17,18,19,20,21,22], that is, the MN12SX density functional is capable of giving HOMO and LUMO energies that allow to verify the agreement with the approximate Koopmans’ theorem. This is not only true because the $J_{HL}$ values are almost zero, but due to the fact that the ΔSL descriptor, which relates to the difference between the LUMO of the neutral and the HOMO of the anion, is also close to zero. Indeed, these values cannot be exactly equal to zero, but the small differences mean that errors in the prediction of the global reactivity descriptors will be negligible. Moreover, it can be seen from Table 1 that the MN12SX density functional predicts negative values for the LUMO energies, which will represent positive values of the electron affinity A.

The KID procedure has its foundations on the behavior of the four descriptors $J_{I}$, $J_{A}$, $J_{HL}$ and ΔSL: The closer they are to zero, the better agreement of a density functional in giving accurate Conceptual DFT descriptors calculated only from the HOMO and LUMO. This allows to avoid the calculation of the energies of the cation and anion species which, being open systems, are more difficult to converge than the parent neutral molecule, which is inconvenient when studying large systems like those considered in this study.

By taking into account the KID procedure presented in our previous works together with the finite difference approximation, the global reactivity descriptors can be expressed as [5,6,50,51,52]:

(1) $$\mathrm{Electronegativity}\;\chi =-\frac{1}{2}\left(I+A\right)\approx \frac{1}{2}\left(\epsilon_{L}+\epsilon_{H}\right)$$

(2) $$\text{Global Hardness}\;\eta =\left(I-A\right)\approx \left(\epsilon_{L}-\epsilon_{H}\right)$$

(3) $$\mathrm{Electrophilicity}\;\omega =\frac{\mu^{2}}{2\eta }=\frac{{\left(I+A\right)}^{2}}{4(I-A)}\approx \frac{{\left(\epsilon_{L}+\epsilon_{H}\right)}^{2}}{4(\epsilon_{L}-\epsilon_{H})}$$

(4) $$\text{Electrodonating Power}\;\omega^{-}=\frac{{\left(3I+A\right)}^{2}}{16(I-A)}\approx \frac{{\left(3\epsilon_{H}+\epsilon_{L}\right)}^{2}}{16\eta }$$

(5) $$\text{Electroaccepting}\;\omega^{+}=\frac{{\left(I+3A\right)}^{2}}{16(I-A)}\approx \frac{{\left(\epsilon_{H}+3\epsilon_{L}\right)}^{2}}{16\eta }$$

(6) $$\text{Net Electrophilicity}\;\Delta \omega^{\pm }=\omega^{+}-\left(-\omega^{-}\right)=\omega^{+}+\omega^{-}$$

where $\epsilon_{H}$ and $\epsilon_{L}$ are the energies of the HOMO and LUMO, respectively.

According to our previous discussion, the results for the global reactivity descriptors based on the values of the HOMO and LUMO energies calculated with the MN12SX density functional are presented in Table 2.

As expected from the molecular structure of this species, its electrodonating ability is more important that its electroaccepting character. However, when comparing the values of the global descriptors for each of the peptides, it can be seen that there are not significant differences between them.

### 4.1. Local Reactivity Descriptors Calculation

Applying the same ideas as before, the definitions for the local reactivity descriptors will be [5,6,53,54,55,56,57,58,59,60]:

(7) $$\text{Nucleophilic Fukui Function}\;f^{+}\left(r\right)=\rho_{N+1}\left(r\right)-\rho_{N}\left(r\right)$$

(8) $$\text{Electrophilic Fukui Function}\;f^{-}\left(r\right)=\rho_{N}\left(r\right)-\rho_{N-1}\left(r\right)$$

(9) $$\text{Dual Descriptor}\;\Delta f\left(r\right)={\left(\frac{\partial \;f(r)}{\partial \;N}\right)}_{\upsilon (r)}$$

(10) $$\text{Nucleophilic Parr Function}\;P{}^{-}\left(r\right)=\rho_{s}^{rc}\left(r\right)$$

(11) $$\text{Electrophilic Parr Function}\;P{}^{+}\left(r\right)=\rho_{s}^{ra}\left(r\right)$$

where $\rho_{N+1}\left(r\right)$, $\rho_{N}\left(r\right)$, and $\rho_{N-1}\left(r\right)$ are the electronic densities at point r for a system with N+1, *N*, and N−1 electrons, respectively, and $\rho_{s}^{rc}\left(r\right)$ and $\rho_{s}^{ra}(r$) are related to the atomic spin density (ASD) at the **r** atom of the radical cation or anion of a given molecule, respectively [61].

**Figure 2:**
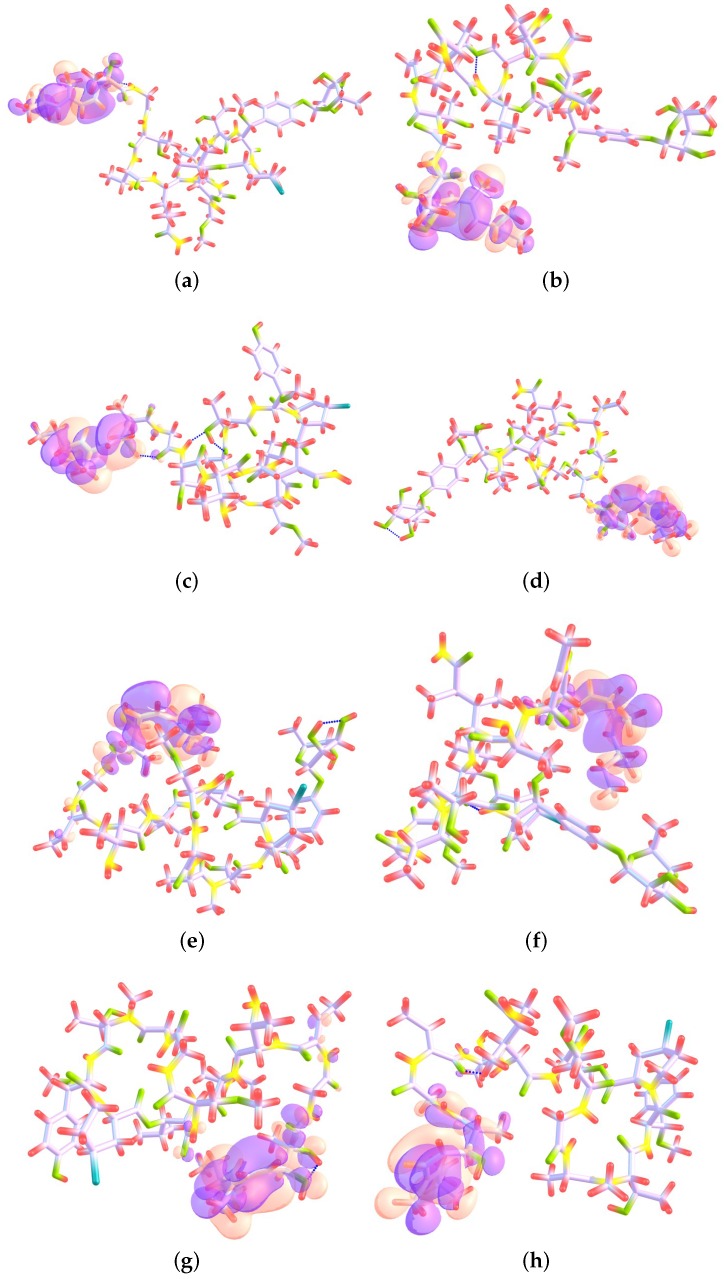
Electrophilic Fukui functions $f^{-}\left(r\right)$ for (**a**) Mirabamide A; (**b**) Mirabamide B; (**c**) Mirabamide C; (**d**) Mirabamide D; (**e**) Mirabamide E; (**f**) Mirabamide F; (**g**) Mirabamide G and (**h**) Mirabamide H—This graphic has been plotted following the ChemCraft style where the following colors have been assigned to the atoms: C: lilac, H: red, N: yellow, O: green and Cl: blue, and orange and purple are assigned to positive and negative surfaces, respectively.

**Figure 3:**
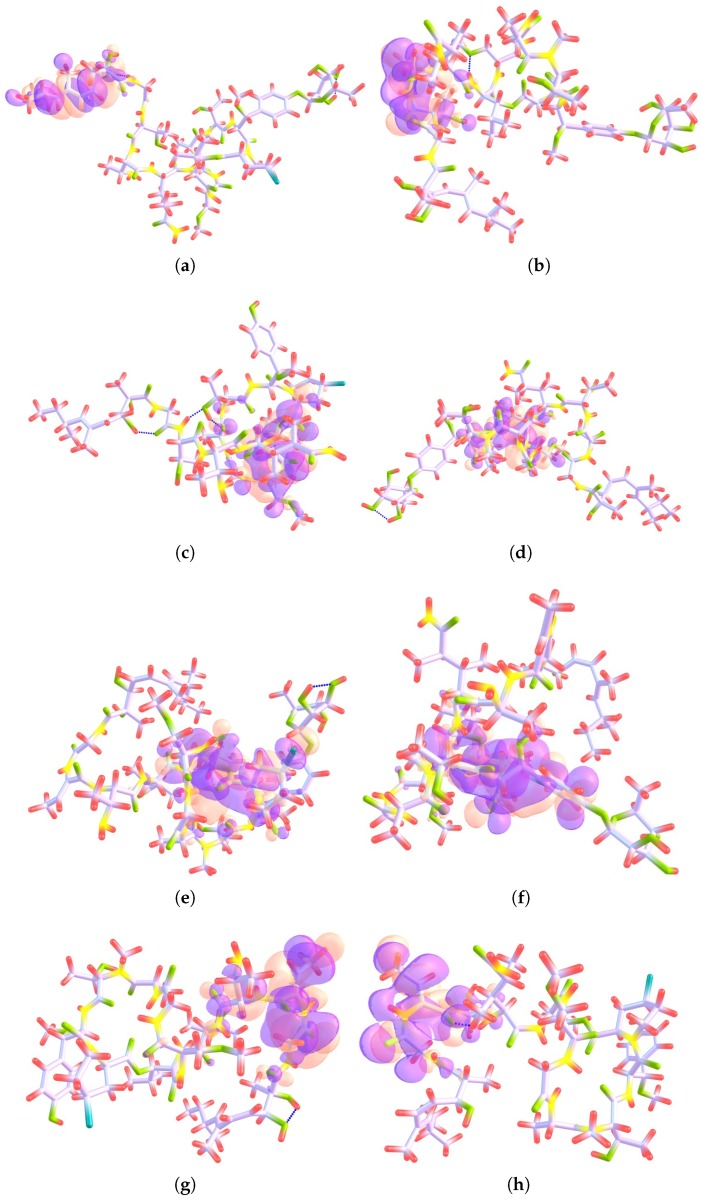
Nucleophilic Fukui functions $f^{+}\left(r\right)$ for (**a**) Mirabamide A; (**b**) Mirabamide B; (**c**) Mirabamide C; (**d**) Mirabamide D; (**e**) Mirabamide E; (**f**) Mirabamide F; (**g**) Mirabamide G and (**h**) Mirabamide H–This graphic has been plotted following the ChemCraft style where the following colors have been assigned to the atoms: C: lilac, H: red, N: yellow, O: green and Cl: blue, and orange and purple are assigned to positive and negative surfaces, respectively.

The Electrophilic Fukui functions $f^{-}\left(r\right)$ and Nucleophilic Fukui functions $f^{+}\left(r\right)$ for the Mirabamides A–H molecules are shown in Figure 2 and Figure 3, respectively.

As it has been stated by Martínez-Araya in a recent work [58], while the Fukui function is a nice descriptor to understand the local reactivity of the molecules, it can be demonstrated that the Dual Descriptor in its condensed form Δ$f_{k}$ will perform better for the prediction of the preferred sites for the electrophilic and nucleophilic attacks. For this reason, we have decided to present the results for the Condensed Dual Descriptor Δ$f_{k}$ as calculated from either Mulliken Population Analysis (M) or Natural Population Analysis (N) in comparison with the Nucleophilic Parr Function $P_{k}^{+}$ and Electrophilic Parr Function $P_{k}^{-}$ proposed by Domingo et al. [59,60], considering atomic spin densities coming from the mentioned Mulliken Population Analysis (M) or from Hirshfeld Population Analysis (H).

The results for the calculation of these local reactivity descriptors for the Mirabamides A to H are presented in Table 3, Table 4, Table 5, Table 6, Table 7, Table 8, Table 9 and Table 10, respectively. It must be noted that we are presenting only the results for those atomic sites where the Δ$f_{k}$ (which is itself multiplied by 100) are greater than one. Also, the H atoms are not shown.

As can be seen from the results in Table 3, C104 will be the site for the nucleophilic attack while C105 will be the preferred site for electrophilic attack during a chemical reaction involving Mirabamide A. By considering the results in Table 4, C89 and C91 will be the sites for the nucleophilic attack while C106 will be the preferred site for electrophilic attack during a chemical reaction involving Mirabamide B. From Table 5, C54 will be the site for the nucleophilic attack while C97 will be the preferred site for electrophilic attack during a chemical reaction involving Mirabamide C. For the case of the Mirabamide D in Table 6, C56 will be the site for the nucleophilic attack while C106 will be the preferred site for electrophilic attack. From the results in Table 7, C47 will be the site for the nucleophilic attack while C106 will be the preferred site for electrophilic attack during a chemical reaction involving Mirabamide E. In the case of Mirabamide F, we can see from Table 8 that C43 will be the site for the nucleophilic attack while C105 will be the preferred site for electrophilic attack during a chemical reaction involving Mirabamide F. Considering the results in Table 9, C84 and C86 will be the sites for the nucleophilic attack while C96 will be the preferred site for electrophilic attack during a chemical reaction involving Mirabamide G. Lastly, from the results in Table 10, C83 will be the site for the nucleophilic attack while C95 will be the preferred site for electrophilic attack during a chemical reaction involving Mirabamide H.

According to the results from Table 3, Table 4, Table 5, Table 6, Table 7, Table 8, Table 9 and Table 10, the local reactivity descriptors calculated from the different formulations are able to recognize the nucleophilic and electrophilic sites for chemical reactivity with great accuracy. Moreover, there is an impressive agreement between the results coming from the Condensed Dual Descriptor Δ$f_{k}$ and the Nucleophilic and Electrophilic Parr Functions $P_{k}^{+}$ and $P_{k}^{-}$, which means that their use in this and future works related to the study of therapeutic peptides will have an increased chance of success.

### 4.2. Calculation of the p$K_{a}$ of the Peptides

We have recently presented a study of the computational prediction of the p$K_{a}$s of small peptides through Conceptual DFT descriptors [12]. In that work, we concluded that the relationship p$K_{a}$ = 16.3088 − 0.8268η could be a valuable starting point for the prediction of the p$K_{a}$ of larger peptides of interest for the development of AGE inhibitors.

At biological pH, the Mirabamides A–H are neutral molecules, and we considered them in that state for our p$K_{a}$ calculations. Following the methodology or our previous work, we have considered the optimized molecular structure of each of the conformers and we have applied the mentioned relationship to the calculation of the p$K_{a}$s of the Mirabamide A–H molecules, with the η values presented in Table 2 being the results as follows in Table 11:

From these results it can be seen that our methodology is able to distinguish between the p$K_{a}$ value of every peptide in spite of the small differences between them. These values can be seen in the context of our previous study [12], and we believe that they could be of interest when designing pharmaceutical drugs starting from these peptides and enabling an explanation of the mechanisms of action and the drug delivery procedures.

### 4.3. Quantification of the AGEs Inhibitor Ability

The Maillard reaction between a reducing carbonyl and the amino group of a peptide or protein leads to the formation of a Schiff base which, through a series of steps, renders different molecules known as Advanced Glycation Endproducts or AGEs. It is believed that the presence of these AGEs is one of the main reasons for the developing of some diseases, such as Diabetes, Alzheimer and Parkinson [62].

Among several strategies that have been considered for the prevention of the formation of AGEs, it is worth mentioning the use of compounds presenting amino groups in their structure capable of interacting with the reducing carbonyl of carbohydrates and being competitive with the amino acids, peptides and proteins present in our body. Many compounds have been devised as drugs to achieve this goal and, to name a few, we can include Pyridoxamine, Aminoguanidine, Carnosine, Metformin, Pioglitazone and Tenilsetam [63,64].

It can be proposed that peptides having amino and amido groups could be thought as potential therapeutic drugs for preventing the formation of AGEs, because they could react in the Maillard reaction with reducing carbohydrates before the peptides and proteins of our body. Although this a merely speculative proposal, we believe that it is worth exploring this possibility by following a methodology we presented earlier. In a previous work, we have studied the ability of a group of proposed molecules to act as inhibitors of the formation of AGEs by quantifying their behavior in terms of Conceptual DFT reactivity descriptors [13]. It was concluded that the key factor in the study of the chemical reactivity of the potential AGEs’ inhibitors was on their nucleophilic character, and although there are several definitions of nucleophilicity [65], our results suggested that the inverse of the net electrophilicity $\Delta \omega^{\pm }$ could be a good definition for the nucleophilicity N. On the basis of the mentioned analysis, we were able to find some qualitative trends for the studied molecular systems.

In this work, we will extend this correlation to the Mirabamide A–H peptides in order to see if they can be considered as precursors of therapeutic drugs for the inhibition of the formation of AGEs, besides their known activity as HIV-Inhibitory peptides. As the model chemistry employed in both works is the same, the comparison is straightforward:

Aminoguanidine>Metformin>Carnosine>Tenilsetam>Mirabamide G>Mirabamide C≈Mirabamide D>Pyridoxamine>Mirabamide B>Mirabamide A≈Mirabamide F>Mirabamide E>Pioglitazone

This qualitative trend is representative of the known pharmacological properties of the studied AGEs inhibitors [63,64] and it can be seen that the Mirabamide A–H possess AGEs Inhibitor Abilities similar to that of Pyridoxamine, with some of them (Mirabamide G, Mirabamide C and Mirabamide D) performing better, while the others present lower values of the AGEs Inhibitor Ability.

### 4.4. Bioactivity Scores

When considering a given molecular system as a potential therapeutic drug, it is customary to check if the considered species follows the Lipinski Rule of Five, which is used to predict whether a compound has or not has a drug-like character [66]. The molecular properties related to the drug-like character were calculated with the aid of the MolSoft and Molinspiration software and are presented in Table 12, where miLog*P* represents the octanol/water partition coefficient, TPSA is the molecular polar surface area, natoms is the number of atom of the molecule, nON and nOHNH are the number of hydrogen bond acceptors and hydrogen bond donors respectively, nviol is the number of violations of the Lipinski Rule of Five, nrotb is the number of rotatable bonds, volume is the molecular volume, and MW is the molecular weight of the studied system.

However, what the Lipinski Rule of Five really measures is the oral bioavailability of a potential drug, because this is desired property for a molecule having drug-like character. Indeed, this criteria cannot be applied to peptides, even when they are small, as we can see from Table 12, due to the inherent molecular weight and number of hydrogen bonds.

In a more recent work, Martin [67] has developed what she called “A Bioavailability Score” (ABS) for avoiding these problems. The rule for the ABS established that the Bioavailability Score for neutral organic molecules must be 0.55 if they pass the Lipinski Rule of Five and 0.170 if they fail. The ABS value for all the Mirabamides A–H considered in this work have been calculated by using the ChemDoodle software, and the results were equal to 0.170 for all of them.

A different approach was then followed by considering similarity searches in the chemical space of compounds with structures that can be compared to those that are being studied and with known pharmacological properties.

As has been mentioned in the Settings and Computational Methods section, this task can be accomplished using the online Molinspiration software for the prediction of the bioactivity score for different drug targets (GPCR (G protein-coupled receptor) ligands, kinase inhibitors, ion channel modulators, enzymes and nuclear receptors). The results are named Bioactivity Scores and the values for the Mirabamides A–H are presented in Table 13.

These bioactivity scores for organic molecules can be interpreted as active (when the bioactivity score is >0), moderately active (when the bioactivity score lies between −5.0 and 0.0) and inactive (when the bioactivity score <−5.0). All the Mirabamides A–H were found to be moderately bioactive towards all the enzymes considered for the study.

As the IC${}_{50}$ values for the HIV-Inhibitory action of the peptides are available from References [10,11], we considered that it was worth verifying if a linear relationship exists between the IC${}_{50}$ values and the global reactivity descriptors presented in Table 2. However, it was not possible to find such a relationship. This can be explained by considering that half of the results came from different experimental studies.

## 5. Conclusions

In this paper we have presented the results of a study of the chemical reactivity of a group of peptides with therapeutic potential, Mirabamides A–H, based on the Conceptual DFT as a tool to explain the molecular interactions.

It must be remarked that the tools used did not generate a huge error despite the relative low computational they have, which is itself a good advantage.

The knowledge of the values of the global and local descriptors of the molecular reactivity of the Mirabamides A–H peptides studied could be useful in the development of new drugs based on these compounds.

In a similar manner, the p$K_{a}$ values for each of the therapeutic peptides have been predicted by resorting to values of the chemical hardness following a previously proposed methodology, and the information that resulted would be helpful in understanding not only the chemical reactivity, but other important properties, such as the water solubility.

A point of special interest has been the quantification of the ability of each peptide to act as an inhibitor in the formation of AGEs, and this could be of importance for the design of medicines for fighting diseases like Diabetes, Alzheimer’s or Parkinson’s.

Finally, the molecular properties related to bioavailability have been predicted using different methodologies already described in the literature, and the descriptors used for the quantification of the bioactivity allowed the characterization of the studied peptides as moderately bioactive towards several ligands and enzymes.

## Figures and Tables

**Table 1:** Electronic energies of the neutral, positive, and negative molecular systems (in au) of the Mirabamides A–H; the HOMO, LUMO, and SOMO orbital energies (in eV); and the $J_{I}$, $J_{A}$, $J_{HL}$ and ΔSL descriptors calculated with the MN12SX density functional and the Def2TZVP basis set using water as the solvent simulated with the SMD parametrization of the IEF-PCM model.

Molecule	Eo	E+	E−	HOMO	LUMO	SOMO	$J_{I}$	$J_{A}$	$J_{\mathrm{HL}}$	ΔSL
Mirabamide A	−5856.10	−5855.88	−5856.15	−6.36	−1.17	−1.19	0.00	0.01	0.01	0.02
Mirabamide B	−5799.59	−5799.37	−5799.63	−6.10	−1.09	−1.07	0.02	0.01	0.03	0.02
Mirabamide C	−5321.42	−5321.19	−5321.45	−6.26	−0.99	−0.99	0.02	0.00	0.02	0.00
Mirabamide D	−5396.70	−5396.47	−5396.74	−6.19	−1.03	−1.03	0.02	0.00	0.02	0.00
Mirabamide E	−5779.75	−5779.52	−5779.79	−6.07	−1.21	−1.18	0.02	0.01	0.02	0.03
Mirabamide F	−5704.64	−5704.42	−5704.68	−6.13	−1.06	−1.07	0.02	0.00	0.02	0.01
Mirabamide G	−5245.09	−5244.86	−5245.12	−6.16	−0.96	−0.94	0.02	0.01	0.02	0.02
Mirabamide H	−5169.97	−5169.74	−5170.02	−6.13	−1.23	−1.22	0.02	0.01	0.02	0.02

**Table 2:** Global reactivity descriptors of the Mirabamides A–H molecules calculated with the MN12SX density functional with the Def2TZVP basis set and the SMD solvation model using water as the solvent.

Molecule	Electronegativity (χ)	Chemical Hardness (η)	Electrophilicity (ω)
Mirabamide A	3.6281	4.9155	1.3389
Mirabamide B	3.5956	5.0132	1.2894
Mirabamide C	3.6227	5.2665	1.2460
Mirabamide D	3.6066	5.1555	1.2615
Mirabamide E	3.6397	4.8640	1.3617
Mirabamide F	3.5969	5.0654	1.2771
Mirabamide G	3.5582	5.1968	1.2181
Mirabamide H	3.6822	4.8970	1.3844
	**Electrodonating** **Power** **(** $\omega^{-}$ **)**	**Electroaccepting** **Power** **(** $\omega^{+}$ **)**	**Net Electrophilicity** **(** $\Delta \omega^{\pm }$ **)**
Mirabamide A	4.7992	1.1711	5.9702
Mirabamide B	4.6900	1.0944	5.7843
Mirabamide C	4.6324	1.0097	5.6421
Mirabamide D	4.6486	1.0420	5.6905
Mirabamide E	4.8473	1.2077	6.0550
Mirabamide F	4.6692	1.0723	5.7415
Mirabamide G	4.5401	0.9819	5.5220
Mirabamide H	4.9160	1.2338	6.1498

**Table 3:** Local reactivity descriptors for the Mirabamide A molecule calculated with the MN12SX density functional with the Def2TZVP basis set and the SMD solvation model using water as the solvent: Condensed Dual Descriptor Δ$f_{k}$, Nucleophilic Parr Function $P_{k}^{+}$ and Electrophilic Parr Function $P_{k}^{-}$; M stands for Mulliken Population Analysis, N corresponds to Natural Population Analysis and H means Hirshfeld Population Analysis.

Atom	Δ$f_{k}$ (M)	Δ$f_{k}$ (N)	$P_{k}^{+}$ (M)	$P_{k}^{-}$ (M)	$P_{k}^{+}$ (H)	$P_{k}^{-}$ (H)
26 O	−1.79	−0.50	0.0269	0.0370	0.0424	0.0408
101 C	2.04	2.88	−0.0058	−0.0344	0.0340	0.0121
104 C	10.28	9.54	0.1742	−0.0281	0.1833	0.0581
105 C	−9.00	−7.61	0.0390	0.1956	0.0881	0.1629
108 C	−1.47	−0.27	0.0087	0.0208	0.0230	0.0287

**Table 4:** Local reactivity descriptors for the Mirabamide B molecule calculated with the MN12SX density functional with the Def2TZVP basis set and the SMD solvation model using water as the solvent: Condensed Dual Descriptor Δ$f_{k}$, Nucleophilic Parr Function $P_{k}^{+}$ and Electrophilic Parr Function $P_{k}^{-}$; M stands for Mulliken Population Analysis, N corresponds to Natural Population Analysis and H means Hirshfeld Population Analysis.

Atom	Δ$f_{k}$ (M)	Δ$f_{k}$ (N)	$P_{k}^{+}$ (M)	$P_{k}^{-}$ (M)	$P_{k}^{+}$ (H)	$P_{k}^{-}$ (H)
18 O	2.98	5.26	0.0367	0.0000	0.0373	0.0000
21 O	12.11	13.61	0.1365	0.0000	0.1426	0.0000
26 O	−3.06	−3.51	0.0000	0.0363	0.0000	0.0365
31 N	1.70	2.03	0.0074	0.0000	0.0190	0.0000
36 N	1.17	−0.64	−0.0481	0.0000	0.0269	0.0000
82 C	5.22	2.97	0.0540	0.0000	0.0521	0.0000
85 C	12.40	5.97	0.1118	0.0000	0.1064	0.0000
89 C	25.07	22.13	0.3306	0.0000	0.2286	0.0000
91 C	25.31	18.18	0.3485	0.0000	0.2251	0.0000
92 C	2.01	−0.99	−0.0317	0.0000	0.0308	0.0000
94 C	2.47	0.83	0.0308	0.0000	0.0247	0.0000
100 C	−1.16	1.01	0.0000	−0.0143	0.0000	0.0222
102 C	−16.25	−15.76	0.0000	0.2328	0.0000	0.1676
103 C	−8.25	−1.92	0.0000	0.0195	0.0000	0.0823
104 C	−23.40	−17.03	0.0000	0.2363	0.0000	0.1973
105 C	−1.27	2.50	0.0000	−0.0113	0.0000	0.0335
106 C	−32.17	−26.77	0.0000	0.4139	0.0000	0.3301
107 C	−2.47	−0.42	0.0000	0.0230	0.0000	0.0315
109 C	−1.71	1.68	0.0002	0.0282	0.0001	0.0542

**Table 5:** Local reactivity descriptors for the Mirabamide C molecule calculated with the MN12SX density functional with the Def2TZVP basis set and the SMD solvation model using water as the solvent: Condensed Dual Descriptor Δ$f_{k}$, Nucleophilic Parr Function $P_{k}^{+}$ and Electrophilic Parr Function $P_{k}^{-}$; M stands for Mulliken Population Analysis, N corresponds to Natural Population Analysis and H means Hirshfeld Population Analysis.

Atom	Δ$f_{k}$ (M)	Δ$f_{k}$ (N)	$P_{k}^{+}$ (M)	$P_{k}^{-}$ (M)	$P_{k}^{+}$ (H)	$P_{k}^{-}$ (H)
4 O	2.28	3.61	0.0347	0.0000	0.0379	0.0000
7 O	17.48	16.50	0.1732	0.0000	0.1830	0.0000
15 O	2.40	3.70	0.0222	0.0000	0.0246	0.0000
22 O	−1.70	−2.56	0.0000	0.0203	0.0000	0.0237
27 N	5.60	3.77	0.0116	0.0000	0.0593	0.0000
31 N	1.56	1.51	0.0097	0.0000	0.0167	0.0000
41 C	2.57	0.43	0.0231	0.0000	0.0337	0.0000
43 C	5.37	6.75	0.1091	0.0000	0.0757	0.0000
45 C	3.98	0.12	0.0036	0.0000	0.0404	0.0000
52 C	1.68	1.44	0.0105	0.0000	0.0170	0.0000
54 C	36.46	24.72	0.4387	0.0000	0.2865	0.0000
61 C	2.57	0.33	0.0312	0.0000	0.0526	0.0000
74 C	5.99	3.60	0.0814	0.0000	0.0513	0.0000
93 C	−17.66	−17.46	0.0000	0.2636	0.0000	0.1817
94 C	−6.66	−0.48	0.0000	−0.0190	0.0000	0.0562
95 C	−25.33	−18.94	0.0000	0.2674	0.0000	0.2175
96 C	−1.60	2.92	0.0000	−0.0046	0.0000	0.0439
97 C	−33.20	−28.08	0.0000	0.4218	0.0000	0.3425
99 C	−1.20	2.14	0.0000	0.0233	0.0000	0.0517
100 C	−2.87	−0.54	0.0000	0.0368	0.0000	0.0439

**Table 6:** Local reactivity descriptors for the Mirabamide D molecule calculated with the MN12SX density functional with the Def2TZVP basis set and the SMD solvation model using water as the solvent: Condensed Dual Descriptor Δ$f_{k}$, Nucleophilic Parr Function $P_{k}^{+}$ and Electrophilic Parr Function $P_{k}^{-}$; M stands for Mulliken Population Analysis, N corresponds to Natural Population Analysis and H means Hirshfeld Population Analysis.

Atom	Δ$f_{k}$ (M)	Δ$f_{k}$ (N)	$P_{k}^{+}$ (M)	$P_{k}^{-}$ (M)	$P_{k}^{+}$ (H)	$P_{k}^{-}$ (H)
1 O	1.29	2.19	−0.0009	0.0000	0.0266	0.0000
3 O	4.05	6.90	0.0575	0.0000	0.0663	0.0000
7 O	15.21	15.15	0.1317	0.0000	0.1416	0.0000
25 O	−1.21	−1.80	0.0000	0.0148	0.0000	0.0155
30 N	6.69	3.82	0.0218	0.0000	0.0764	0.0000
44 C	1.13	1.33	0.0162	0.0000	0.0107	0.0000
45 C	2.71	1.64	0.0275	0.0000	0.0347	0.0000
47 C	10.51	9.80	0.2036	0.0000	0.1360	0.0000
48 C	4.13	−0.50	−0.0126	0.0000	0.0461	0.0000
56 C	35.91	20.68	0.4392	0.0000	0.2739	0.0000
85 C	2.57	0.20	0.0217	0.0000	0.0135	0.0000
100 C	−1.47	1.55	0.0000	−0.0048	0.0000	0.0352
102 C	−15.85	−16.36	0.0001	0.2247	0.0001	0.1596
103 C	−7.09	−0.93	0.0000	0.0042	0.0000	0.0701
104 C	−24.77	−18.19	0.0000	0.2629	0.0000	0.2108
105 C	−1.18	2.47	0.0000	−0.0157	0.0000	0.0283
106 C	−32.26	−26.83	0.0000	0.4080	0.0000	0.3312
107 C	−2.34	−0.36	0.0000	0.0212	0.0000	0.0293
108 C	−1.67	1.81	0.0000	0.0273	0.0000	0.0560
109 C	−1.15	0.15	0.0000	0.0155	0.0000	0.0199

**Table 7:** Local reactivity descriptors for the Mirabamide E molecule calculated with the MN12SX density functional with the Def2TZVP basis set and the SMD solvation model using water as the solvent: Condensed Dual Descriptor Δ$f_{k}$, Nucleophilic Parr Function $P_{k}^{+}$ and Electrophilic Parr Function $P_{k}^{-}$; M stands for Mulliken Population Analysis, N corresponds to Natural Population Analysis and H means Hirshfeld Population Analysis.

Atom	Δ$f_{k}$ (M)	Δ$f_{k}$ (N)	$P_{k}^{+}$ (M)	$P_{k}^{-}$ (M)	$P_{k}^{+}$ (H)	$P_{k}^{-}$ (H)
1 Cl	1.21	2.34	0.0098	0.0000	0.0117	0.0000
2 O	7.00	6.14	0.0226	0.0000	0.0800	0.0000
4 O	18.05	17.88	0.1574	0.0000	0.1865	0.0000
8 O	1.12	2.68	0.0197	0.0001	0.0192	0.0000
24 O	−1.93	−2.03	0.0000	0.0260	0.0000	0.0225
25 O	−2.92	−3.48	0.0000	0.0331	0.0000	0.0335
39 C	3.55	−0.74	−0.0346	0.0000	0.0471	0.0000
40 C	2.74	0.03	0.0175	0.0000	0.0342	0.0000
41 C	1.34	0.20	0.0143	0.0000	0.0125	0.0000
47 C	47.05	32.44	0.6208	0.0000	0.3907	0.0000
56 C	2.34	0.88	0.0288	−0.0003	0.0336	0.0000
57 C	1.30	1.47	0.0195	0.0000	0.0130	0.0000
98 C	−1.50	−0.65	0.0001	0.0156	0.0000	0.0161
100 C	−1.70	1.32	0.0000	0.0042	0.0000	0.0387
102 C	−15.73	−14.81	0.0001	0.2123	0.0001	0.1588
103 C	−9.70	−4.25	0.0001	0.0503	0.0001	0.0967
104 C	−21.41	−14.92	0.0000	0.2162	0.0001	0.1804
105 C	−1.38	2.25	0.0000	−0.0111	0.0000	0.0353
106 C	−28.96	−24.20	0.0002	0.3703	0.0001	0.2972
107 C	−2.56	−0.46	0.0000	0.0274	0.0000	0.0325
108 C	−1.06	1.75	0.0001	0.0174	0.0004	0.0424

**Table 8:** Local reactivity descriptors for the Mirabamide F molecule calculated with the MN12SX density functional with the Def2TZVP basis set and the SMD solvation model using water as the solvent: Condensed Dual Descriptor Δ$f_{k}$, Nucleophilic Parr Function $P_{k}^{+}$ and Electrophilic Parr Function $P_{k}^{-}$; M stands for Mulliken Population Analysis, N corresponds to Natural Population Analysis and H means Hirshfeld Population Analysis.

Atom	Δ$f_{k}$ (M)	Δ$f_{k}$ (N)	$P_{k}^{+}$ (M)	$P_{k}^{-}$ (M)	$P_{k}^{+}$ (H)	$P_{k}^{-}$ (H)
2 O	1.83	2.09	−0.0002	0.0000	0.0182	0.0000
3 O	12.58	13.06	0.1557	0.0000	0.1606	0.0000
4 O	7.22	7.19	0.0594	0.0000	0.0649	0.0000
24 O	−4.19	−4.69	0.0000	0.0494	0.0000	0.0502
25 N	4.63	2.78	0.0150	0.0000	0.0407	0.0000
26 N	1.05	1.03	0.0043	0.0000	0.0260	0.0000
38 C	1.43	−0.47	−0.0101	0.0000	0.0252	0.0000
43 C	25.56	20.56	0.3308	0.0000	0.2266	0.0000
45 C	4.14	0.43	0.0265	0.0000	0.0453	0.0000
46 C	17.37	9.70	0.1869	0.0000	0.1239	0.0000
55 C	1.50	0.91	0.0186	0.0000	0.0156	0.0000
58 C	1.46	1.25	0.0169	0.0000	0.0132	0.0000
66 C	2.41	3.02	0.0221	0.0000	0.0276	0.0000
67 C	1.50	2.17	0.0185	0.0000	0.0203	0.0000
74 C	2.00	2.19	0.0362	0.0000	0.0267	0.0000
75 C	2.25	2.51	0.0392	0.0000	0.0300	0.0000
99 C	−1.18	1.08	0.0000	−0.0094	0.0000	0.0226
101 C	−17.34	−16.63	0.0000	0.2448	0.0000	0.1801
102 C	−8.93	−2.85	0.0000	0.0242	0.0000	0.0824
103 C	−22.46	−16.32	0.0000	0.2238	0.0000	0.1902
104 C	−1.43	2.42	0.0001	−0.0137	0.0001	0.0331
105 C	−31.09	−26.23	0.0000	0.4019	0.0000	0.3214
106 C	−2.76	−0.58	0.0002	0.0278	0.0005	0.0337
107 C	−1.06	1.90	0.0000	0.0196	0.0000	0.0449

**Table 9:** Local reactivity descriptors for the Mirabamide G molecule calculated with the MN12SX density functional with the Def2TZVP basis set and the SMD solvation model using water as the solvent: Condensed Dual Descriptor Δ$f_{k}$, Nucleophilic Parr Function $P_{k}^{+}$ and Electrophilic Parr Function $P_{k}^{-}$; M stands for Mulliken Population Analysis, N corresponds to Natural Population Analysis and H means Hirshfeld Population Analysis.

Atom	Δ$f_{k}$ (M)	Δ$f_{k}$ (N)	$P_{k}^{+}$ (M)	$P_{k}^{-}$ (M)	$P_{k}^{+}$ (H)	$P_{k}^{-}$ (H)
17 O	5.31	7.35	0.0502	0.0001	0.0592	0.0001
18 O	10.57	10.62	0.1287	0.0002	0.1339	0.0001
20 O	−1.74	−2.48	0.0055	0.0263	0.0049	0.0234
21 O	−1.45	0.00	0.0000	0.0186	0.0000	0.0208
28 N	3.69	3.00	0.0170	0.0000	0.0420	0.0000
64 C	1.19	−0.41	0.0170	0.0000	0.0111	0.0000
81 C	11.78	0.00	0.1661	−0.0001	0.1104	0.0000
83 C	11.17	22.77	0.0670	0.0002	0.0882	0.0003
84 C	21.83	−1.85	0.2825	0.0009	0.2041	0.0007
86 C	20.87	−0.58	0.2631	−0.0001	0.1909	0.0000
88 C	−1.01	0.99	0.0004	0.0121	0.0005	0.0107
92 C	−19.65	−3.97	0.0000	0.2798	0.0000	0.2050
93 C	−10.38	2.63	0.0000	0.0335	0.0000	0.0868
94 C	−1.31	−15.06	0.0000	−0.0100	0.0000	0.0347
95 C	−20.37	−26.51	0.0000	0.1902	0.0000	0.1721
96 C	−30.34	0.30	0.0000	0.3993	0.0000	0.3179
98 C	−2.48	1.80	0.0000	0.0335	0.0000	0.0410

**Table 10:** Local reactivity descriptors for the Mirabamide H molecule calculated with the MN12SX density functional with the Def2TZVP basis set and the SMD solvation model using water as the solvent: Condensed Dual Descriptor Δ$f_{k}$, Nucleophilic Parr Function $P_{k}^{+}$ and Electrophilic Parr Function $P_{k}^{-}$; M stands for Mulliken Population Analysis, N corresponds to Natural Population Analysis and H means Hirshfeld Population Analysis.

Atom	Δ$f_{k}$ (M)	Δ$f_{k}$ (N)	$P_{k}^{+}$ (M)	$P_{k}^{-}$ (M)	$P_{k}^{+}$ (H)	$P_{k}^{-}$ (H)
17 O	6.06	7.35	0.0672	0.0003	0.0700	0.0004
18 O	8.62	10.62	0.1056	0.0001	0.1078	0.0001
20 O	−1.56	−2.48	0.0001	0.0193	0.0001	0.0230
28 N	3.30	3.00	0.0140	0.0009	0.0328	0.0007
33 N	−1.35	−1.12	0.0062	0.0279	0.0130	0.0238
80 C	11.08	7.23	0.1338	−0.0002	0.0996	0.0000
82 C	13.09	7.21	0.0740	0.0001	0.1039	0.0000
83 C	29.19	22.77	0.3995	−0.0001	0.2770	0.0000
85 C	16.66	13.06	0.2195	0.0002	0.1554	0.0001
90 C	−0.18	0.00	0.0004	0.0026	0.0004	0.0031
91 C	−19.87	−18.12	0.0005	0.2872	0.0002	0.2052
92 C	−10.07	−3.97	−0.0003	0.0233	0.0001	0.0838
93 C	−1.35	2.63	0.0000	−0.0076	0.0000	0.0392
94 C	−21.32	−15.06	0.0005	0.2032	0.0002	0.1794
95 C	−31.07	−26.51	−0.0002	0.4064	0.0004	0.3225
97 C	−2.47	−0.49	0.0000	0.0332	0.0000	0.0400
98 C	−1.00	1.80	0.0000	0.0192	0.0000	0.0422

**Table 11:** p$K_{a}$s of the Mirabamide A–H molecules.

Molecule	p$K_{a}$
Mirabamide A	12.245
Mirabamide B	12.164
Mirabamide C	11.954
Mirabamide D	12.046
Mirabamide E	12.287
Mirabamide F	12.121
Mirabamide G	12.012
Mirabamide H	12.260

**Table 12:** Molecular properties of the Mirabamides A–H peptides calculated to verify the Lipinski Rule of Five.

Molecule	miLog *P*	TPSA	nAtoms	nON	nOHNH	Nviol	Nrotb	Volume	MW
Mirabamide A	−4.35	576.45	111	38	20	3	29	1447.27	1597.22
Mirabamide B	−2.61	550.43	110	37	18	3	28	1429.74	1580.19
Mirabamide C	−3.64	517.53	101	34	18	3	27	1323.41	1451.08
Mirabamide D	−4.41	576.45	110	38	20	3	29	1433.71	1562.78
Mirabamide E	−3.13	556.23	110	37	19	3	28	1433.02	1579.21
Mirabamide F	−2.19	536.00	109	36	18	3	28	1425.32	1563.21
Mirabamide G	−2.36	497.30	100	33	17	3	26	1309.15	1433.07
Mirabamide H	−1.41	477.07	99	32	16	3	26	1301.46	1417.07

**Table 13:** Bioactivity scores of the Mirabamides A–H molecules calculated on the basis of GPCR Ligand, Ion Channel Modulator, Nuclear Receptor Ligand, Kinase Inhibitor, Protease Inhibitor and Enzyme Inhibitor intteractions.

Molecule	GPCR Ligand	Ion Channel Modulator	Kinase Inhibitor	Nuclear Receptor Ligand	Protease Inhibitor	Enzyme Inhibitor
Mirabamide A	−3.99	−4.04	−4.05	−4.05	−3.95	−3.99
Mirabamide B	−3.99	−4.04	−4.06	−4.05	−3.95	−3.99
Mirabamide C	−3.94	−4.00	−4.01	−4.00	−3.89	−4.05
Mirabamide D	−3.98	−4.03	−4.05	−4.04	−3.94	−3.98
Mirabamide E	−3.99	−4.04	−4.06	−4.05	−3.95	−3.99
Mirabamide F	−3.98	−4.04	−4.06	−4.05	−3.95	−3.98
Mirabamide G	−3.94	−4.00	−4.02	−4.01	−3.89	−3.94
Mirabamide H	−3.93	−4.00	−4.02	−4.01	−3.89	−3.94
